# Expert UK consensus on the definition of high risk of recurrence in HER2-negative early breast cancer: A modified Delphi panel

**DOI:** 10.1016/j.breast.2023.103582

**Published:** 2023-09-17

**Authors:** E.R. Copson, J.E. Abraham, J.P. Braybrooke, D. Cameron, S.A. McIntosh, C.O. Michie, A.F.C. Okines, C. Palmieri, F. Raja, R. Roylance, S. Spensley, on behalf of the Delphi panellists

**Affiliations:** a Cancer Sciences Academic Unit, University of Southampton, Southampton, UK; b Precision Breast Cancer Institute, Department of Oncology, University of Cambridge, Cambridge, UK; c Cambridge University Hospitals NHS Foundation Trust, Cambridge, UK; d University Hospitals Bristol and Weston NHS Foundation Trust, Bristol, UK; e Edinburgh Cancer Centre, Western General Hospital, Edinburgh, UK; f Patrick G Johnston Centre for Cancer Research, Queen's University Belfast, Belfast, UK; g The Royal Marsden NHS Foundation Trust, London, UK; h The Clatterbridge Cancer Centre NHS Foundation Trust, Liverpool, UK; i Department of Molecular and Clinical Cancer Medicine, University of Liverpool, Liverpool, UK; j University College London Hospitals NHS Foundation Trust, London, UK; k North Middlesex University Hospital, North Middlesex University Hospital NHS Trust, London, UK; l NIHR University College London Hospitals Biomedical Research Centre, London, UK; m Musgrove Park Hospital, Somerset NHS Foundation Trust, Taunton, UK

**Keywords:** Early breast cancer, Delphi panel, Recurrence, High risk

## Abstract

**Background:**

There is currently no standardised definition for patients at high risk of recurrence of human epidermal growth factor receptor 2 (HER2)-negative early breast cancer (eBC; stages 1–3) after surgery. This modified Delphi panel aimed to establish expert UK consensus on this definition, separately considering hormone receptor (HR)-positive and triple-negative (TN) patients.

**Methods:**

Over three consecutive rounds, results were collected from 29, 24 and 22 UK senior breast cancer oncologists and surgeons, respectively. The first round aimed to determine key risk factors in each patient subgroup; subsequent rounds aimed to establish appropriate risk thresholds. Consensus was pre-defined as ≥70% of respondents.

**Results:**

Expert consensus was achieved on need to assess age, tumour size, tumour grade, number of positive lymph nodes, inflammatory breast cancer and risk prediction tools in all HER2-negative patients. There was additional agreement on use of tumour profiling tests and biomarkers in HR-positive patients, and pathologic complete response (pCR) status in TN patients. Thresholds for high recurrence risk were subsequently agreed. In HR-positive patients, these included age <35 years, tumour size >5 cm (as independent risk factors); tumour grade 3 (independently and combined with other high-risk factors); number of positive nodes ≥4 (independently) and ≥1 (combined). For TN patients, the following thresholds reached consensus, both independently and in combination with other factors: tumour size >2 cm, tumour grade 3, number of positive nodes ≥1.

**Conclusions:**

The results may be a valuable reference point to guide recurrence risk assessment and decision-making after surgery in the HER2-negative eBC population.

## Highlights

• Definitions for high risk of early breast cancer recurrence lack standardisation
• Key criteria include age, tumour size/grade, nodal status and inflammatory disease
• Risk thresholds, profiling tests and assessment tools differ by biological subtype
• A standardised definition of high risk would improve equity of access to treatment
• Greater standardisation could also inform trial design and economic evaluations

## 1 Introduction

Early breast cancer (eBC; stages 1–3) accounts for over 90% of newly-diagnosed cases of breast cancer within the UK, with human epidermal growth factor receptor 2 (HER2)-negative tumours accounting for 85% of eBC [1,2].^a^ Despite this, there is currently no standardised UK definition of patients at ‘high risk’ of recurrence with HER2-negative eBC after surgery. Identifying high-risk patients in a robust, reproducible way would enable consistency of patient access to treatment, not only to adjuvant chemotherapy but to novel targeted therapies recommended for use in high-risk populations. A clearer definition of high risk could also inform the design of future clinical trials and facilitate comparisons of efficacy and cost-effectiveness across therapies.

Risk assessment in breast cancer has evolved significantly over time. For many years, lymph node status was the only prognostic factor considered [3]. This was gradually supplanted by the Nottingham Prognostic Index (NPI), devised in 1982 and based on nodal status, tumour size and tumour grade [4]. More recently, understanding of the different prognostic implications of hormone receptor (HR) and HER2 status has grown, along with other biomarkers. The prognostic value of Ki-67 status, for example, has been demonstrated in several trials (notably POETIC and WSG-ADAPT HR+/HER2–), although technical difficulties have so far prevented widespread adoption in routine clinical practice [[5], [6], [7]]. These developments have been reflected in successive iterations of the St Gallen International Consensus Guidelines, in the make-up of newer prognostic tools, such as NHS Predict, and in genomic-based tests such as Oncotype DX [[8], [9], [10], [11]]. Clinical practice guidelines from the UK National Institute for Health and Care Excellence (NICE), last updated in 2018, refer to a low, medium/intermediate and high risk of disease recurrence, but do not provide detailed guidance on the criteria for these categories [12,13].

Several targeted therapies have recently demonstrated efficacy in phase 3 trials in treating high-risk HER2-negative breast cancer, notably abemaciclib, ribociclib, olaparib and pembrolizumab [[14], [15], [16], [17]]. However, each of these trials utilised different eligibility criteria in defining high-risk populations. For example, abemaciclib was trialled in HR-positive patients, with inclusion based on nodal status, tumour grade, tumour size and Ki-67 index [14]. Ribociclib – a cyclin-dependent kinase 4 and 6 (CDK4/6) inhibitor like abemaciclib – was also trialled in HR-positive patients in conjunction with endocrine therapy in the adjuvant setting, but in a broader population of patients with American Joint Committee on Cancer (AJCC) stage 2–3 disease, including those with node-negative disease [18]. Meanwhile, the efficacy of olaparib was assessed in a germline breast cancer susceptibility gene 1/2 (*BRCA1/2*)-mutated HER2-negative population stratified by HR status and receipt of neoadjuvant therapy, using different combinations of factors such as nodal status, tumour size, pathologic complete response (pCR) and CPS + EG score [16]. Pembrolizumab was evaluated in the neoadjuvant and adjuvant setting in patients with AJCC stage 2–3 triple-negative (TN) breast cancer [17].

Recognising that the assessment of high risk of recurrence is often multifactorial, the aim of this study was to establish whether there is expert UK consensus on this definition, separately considering HR-positive and TN patients within the HER2-negative population, using a modified Delphi panel design.

The Delphi technique is a well-established method for collecting and building expert consensus, and has been used to establish clinical consensus in the field of breast cancer [[19], [20], [21], [22]]. Such studies include a recent Delphi panel among Italian experts on the definition of high risk in HR-positive, HER2-negative eBC [22]. The Delphi method involves a series of survey rounds, in each of which a group of experts are asked to respond individually to a set of statements or questions. The statements for each round are based on the findings of the previous one, the aim being to achieve consensus (set at a pre-defined percentage agreement threshold) on each statement, or failing this, establish key areas of heterogeneity. Participants are able to see the results of previous rounds, including their own responses, which allows them to reflect on the views of others and adjust their responses as they deem appropriate. However, responses are shared with the broader group anonymously, guarding against the shortcomings of conventional consensus-gathering methods, such as group pressure for conformity and the influence of dominant individuals [23,24].

## 2 Materials and methods

### 2.1 Delphi panellists

An initial pool of 79 breast cancer oncologists and surgeons from around the UK (including clinicians from England, Scotland, Wales and Northern Ireland) were invited by email to participate in the Delphi panel. All invitees had a consultant-level clinical practice within the National Health Service (NHS), including clinical academics. Following the invitation, 45 clinicians accepted to participate in the Delphi panel, of which 29 responded to the Round 1 questionnaire, 24 to Round 2, and 22 to Round 3 (only those who responded to a given round were invited to complete the next round).

One lead clinician (ERC) was invited to guide the development of statements and answer options for each round of the Delphi panel. To avoid potential bias, the lead clinician did not actively participate in the consensus process. The sponsor (10.13039/100004325AstraZeneca) did not actively participate in the consensus process but reviewed the questionnaires to ensure technical accuracy, regulatory compliance and practical applicability of the consensus for the clinical community. No patients were involved in the study; hence, ethical approval was not required.

### 2.2 Study design

The modified Delphi method uses an iterative, anonymised approach to robustly elicit and synthesise responses from participants over up to three sequential rounds of surveys. Each questionnaire round was delivered through a bespoke web application helping to enforce key methodological requirements for Delphi panels, such as preventing retrospective amendments to a questionnaire round. All responses were anonymised, and panellists were able to view their own previous response alongside a chart displaying anonymised summary statistics and free-text comments provided for the preceding round.

Statements comprised free-text, single-choice or numerical formats. Consensus for single-choice questions was set at a pre-defined threshold of ≥70% of respondents, which is considered standard [23]. Consensus was not assessed for free-text or numerical questions.

### 2.3 Questionnaire development

In Round 1, statements were developed using the results from a targeted literature review and guidance from the lead clinician. Full methodology of the targeted literature review is presented in the Supplementary Materials.

Statements for Rounds 2 and 3 were informed by results of the previous rounds. Statements that achieved consensus in Rounds 1 or 2, or were not close to achieving consensus (<60% agreement), were removed from subsequent rounds. Statements that were close to reaching consensus were either restated or rephrased in the next round with a view to building consensus, for example by removing response options that were selected by <15% of participants or providing further context on the question scope. Whether to restate or rephrase a statement was decided based on the distribution of responses in the previous round, and the advice of the lead clinician based on free-text comments provided by panellists.

### 2.4 Questionnaire content

Round 1 aimed to establish the most commonly used timeframes, risk thresholds and specific factors in the assessment of risk in the subpopulations of interest. By way of scoping statements, panellists were asked to indicate which combination of timeframe (2 years, 5 years, 10 years, other) and recurrence threshold (>10%, >20%, >30%, other) they deemed most appropriate when considering high risk of recurrence in HR-positive/HER2-negative and TN eBC patients. Panellists were also asked to provide free-text responses indicating the process by which they would currently assess risk of recurrence, and to indicate whether further stratification, beyond HR status, was needed in the current exercise.

Following these scoping statements, panellists were asked to indicate their support for use of specific criteria in defining high risk in routine clinical practice. They were then asked in which subpopulations the criterion should apply: all HER2-negative patients, HR-positive/HER2-negative patients only, or TN patients only. An opportunity was provided to suggest further criteria or comments.

In Rounds 2 and 3, the objective was to establish thresholds indicative of high risk over a 10-year timeframe for each of the factors retained in Round 1. In each case, participants were asked to consider thresholds independently indicative of high risk, as well as thresholds indicative of high risk when present in combination with other high-risk factors. Thresholds were not queried for binary factors such as pCR and the presence of inflammatory breast cancer. Additional statements were included to query the use of specific tumour profiling tests and risk prediction tools.

Full statements for each round are presented in the Supplementary Materials.

## 3 Results

**Fig. 1:**
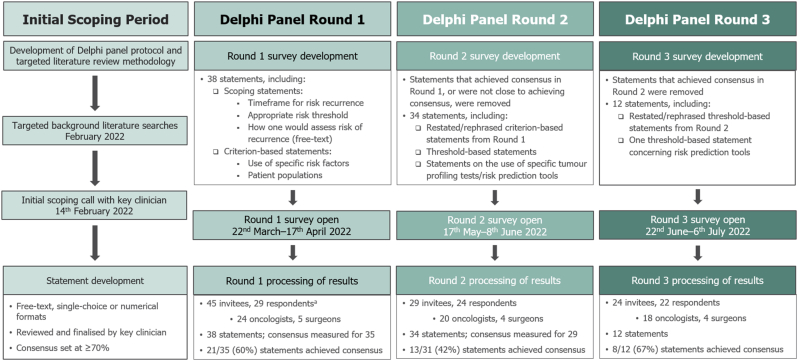
Delphi panel study design. **Footnote:**^a^ From an initial pool of 79 UK-based clinicians contacted, 45 accepted the invitation to participate in the Delphi panel. Of the 29 respondents in Round 1, 22 practise in England, 4 in Scotland, 2 in Wales, and 1 in Northern Ireland, with a consistent split between oncologists and surgeons of roughly 5:1 across all rounds. The breakdown by NHS region among the English clinicians was as follows: 5 respondents practise in London, 5 in the South East, 3 in the East of England, 3 in the North West, 3 in the South West, 2 in the Midlands, and 1 in the North East and Yorkshire.

An outline of the Delphi process and timelines is presented in Fig. 1. Results for the different statements in each round are presented below, categorised by content (scoping statements, risk factors and patient populations, risk thresholds, and tumour profiling tests and risk prediction tools).

### 3.1 Scoping statements (Round 1)

Consensus was not reached on an appropriate timeframe for assessing high risk of recurrence in either subpopulation, with the most frequent response being 10 years for HR-positive/HER2-negative patients (29%, 8/28) and 2 years for TN patients (66%, 19/29; Table 1). There was similar heterogeneity in the selected risk percentage thresholds determined to be ‘high’ in relation to each timeframe.

**Table 1:** Responses to Round 1 scoping statements.

**Statement**	**Outcome**	**Response breakdown (%, n/N)**^**a**^
Please indicate which of the following timeframes is most appropriate when considering ‘high’ risk of recurrence in a patient with HR-positive/HER2-negative eBC.	No consensus reached	2 years (25%, 7/28) 5 years (25%, 7/28) 10 years (29%, 8/28) Other (21%, 6/28)^b^ *No response: 1*

With respect to your answer to the previous question, please indicate which of the following recurrence thresholds most closely matches your perception of ‘high’ risk of recurrence in a patient with HR-positive/HER2-negative eBC.		
*Answer to previous statement: 2 years*	No consensus reached	>10% risk (43%, 3/7) >20% risk (43%, 3/7) >30% risk (14%, 1/7) Other (0) *No response: 0*
*Answer to previous statement: 5 years*	**Agreement**	>10% risk (29%, 2/7) **>20% risk (71%, 5/7)** >30% risk (0) Other (0) *No response: 0*
*Answer to previous statement: 10 years*	No consensus reached	>10% risk (0) >20% risk (50%, 4/8) >30% risk (50%, 4/8) Other (0) *No response: 0*
*Answer to previous statement: Other*	No consensus reached	>10% risk (0) >20% risk (67%, 4/6) >30% risk (17%, 1/6) Other (17%, 1/6) *No response: 0*

Please indicate which of the following timeframes is most appropriate when considering ‘high’ risk of recurrence in a patient with TN eBC.	No consensus reached	2 years (66%, 19/29) 5 years (31%, 9/29) 10 years (0) Other (3%, 1/29)^b^ *No response: 0*

With respect to your answer to the previous question, please indicate which of the following recurrence thresholds most closely matches your perception of ‘high’ risk of recurrence in a patient with TN eBC.		
*Answer to previous statement: 2 years*	No consensus reached	>10% risk (37%, 7/19) >20% risk (37%, 7/19) >30% risk (21%, 4/19) Other (5%, 1/19) *No response: 0*
*Answer to previous statement: 5 years*	No consensus reached	>10% risk (22%, 2/9) >20% risk (56%, 5/9) >30% risk (22%, 2/9) Other (0) *No response: 0*
*Answer to previous statement: 10 years*	N/A	N/A
*Answer to previous statement: Other*	**Agreement**	>10% risk (0) **>20% risk (100%, 1/1)**^**c**^ >30% risk (0) Other (0) *No response: 0*

Within the HR-positive/HER2-negative and TN eBC populations, key factors needed to determine risk of recurrence after surgery may differ between patient subgroups (e.g. depending on whether patients received neoadjuvant or adjuvant chemotherapy). On this basis:		
Please indicate whether further stratification of the definitions for high risk of recurrence (including the use of different risk factors and/or risk thresholds for key patient subgroups) is needed in clinical practice.	**Agreement**	No further stratification (11%, 3/28) **Further stratification (89%, 25/28)** *No response: 1*

**Footnotes:**^a^ Percentage agreement was calculated as a proportion of the number of respondents to a given question (N), i.e. excluding instances of ‘No response’. ^b^ Where ‘Other’ was selected, panellists were invited to specify their preferred timeframe. ^c^ The 100% agreement is an artefact of the single respondent having selected the ‘Other’ option in the upstream branching logic and does not represent meaningful consensus. **Abbreviations:** eBC: early breast cancer; HER2: human epidermal growth factor receptor 2; HR: hormone receptor; TN: triple-negative.

Answers provided to free-text statements did not warrant inclusion of any additional risk factors or further stratification of the definition for high risk of recurrence, beyond HR status.

### 3.2 Risk factors and patient populations (Rounds 1–2)

**Fig. 2:**
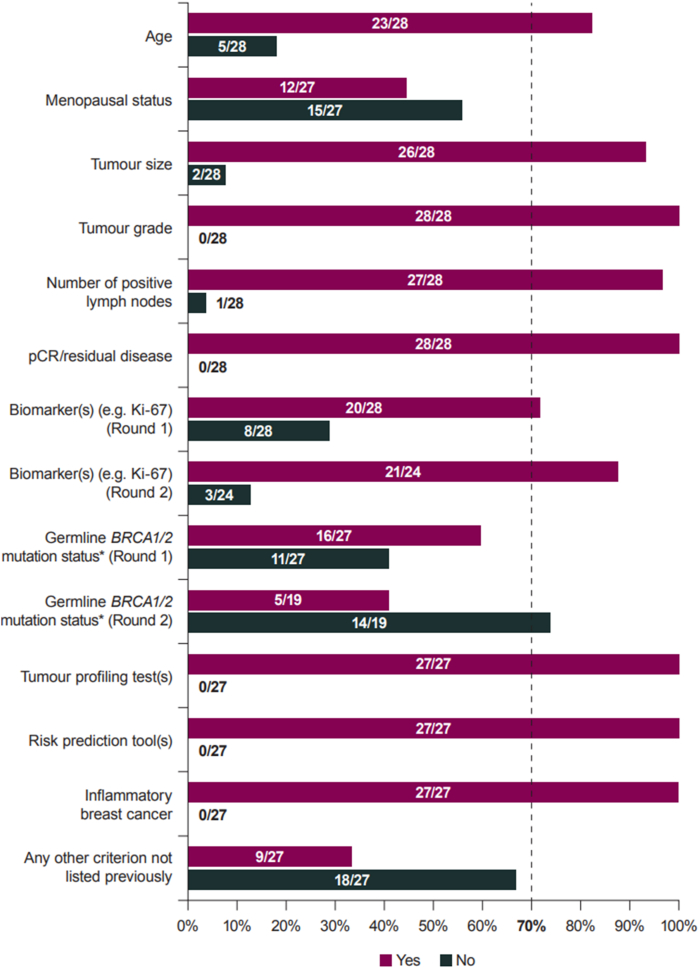
Responses to criterion-based statements: risk factors (Rounds 1–2) **Footnote:** Percentage agreement was calculated as a proportion of the number of respondents to a given question, i.e. excluding instances of ‘No response’. **Abbreviations:***BRCA1/2*: breast cancer susceptibility gene 1/2; pCR: pathologic complete response.

Overall, 9/11 criterion-based statements reached consensus in Round 1 (>70% ‘Yes’): age, tumour size, tumour grade, number of positive nodes, pCR/residual disease, biomarker(s), tumour profiling test(s), risk prediction tool(s) and inflammatory breast cancer (Fig. 2; Table S1). The remaining 2/11 criteria did not achieve consensus, including *BRCA1/2* status (59% ‘Yes’) and menopausal status (56% ‘No’).

Given the small margin to consensus, *BRCA1/2* status was restated in Round 2, upon which there was consensus that it should not be used to define high risk of recurrence (74% ‘No’). The statement relating to use of biomarker(s) was also revised and restated in Round 2, despite reaching consensus, as free-text notes indicated misunderstanding on the scope of the statement. As in Round 1, there was consensus that one or more biomarker(s) should be used (88% ‘Yes’), though free-text notes did not show support for any specific biomarkers beyond 10.13039/501100014832HR and HER2 status. In particular, opinion was divided on the use of Ki-67 as a marker of recurrence risk, with many respondents highlighting its lack of reliability and the absence of standardised risk thresholds to guide decision-making.

**Fig. 3:**
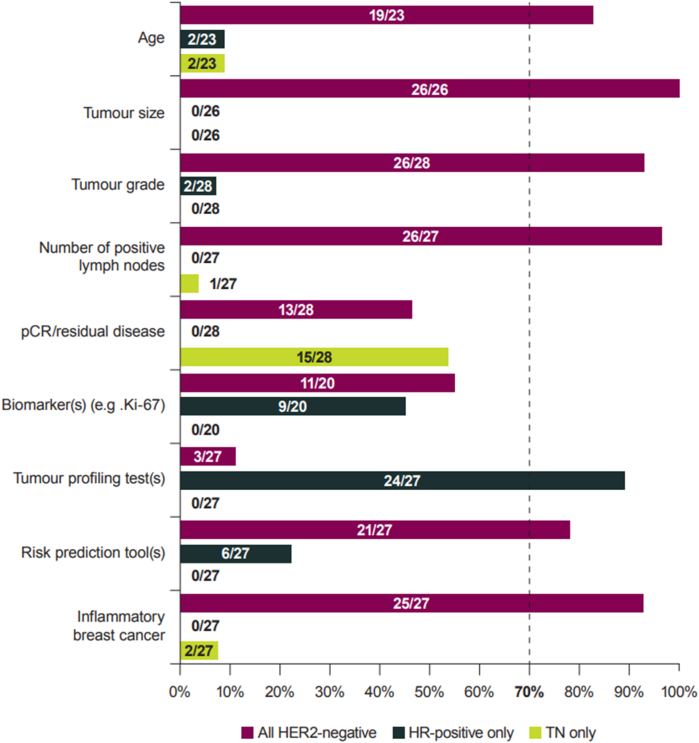
Responses to criterion-based statements: patient populations (Round 1) **Footnote:** Percentage agreement was calculated as a proportion of the number of respondents to a given question, i.e. excluding instances of ‘No response’. **Abbreviations:** HER2: human epidermal growth factor receptor 2; HR: hormone receptor; pCR: pathologic complete response; TN: triple-negative.

Among the nine criteria that reached consensus for use, 7/9 factors achieved consensus on applicable population (Fig. 3; Table S2). Six of these received consensus for use in all HER2-negative patients: age, tumour size, tumour grade, number of positive nodes, risk prediction tool(s) and inflammatory breast cancer. Participants agreed that tumour profiling test(s) and biomarkers should be used to determine high risk of recurrence in HR-positive/HER2-negative patients only. Conversely, there was consensus that pCR/residual disease should be used in assessing TN patients, but this criterion failed to reach consensus for the HR-positive/HER2-negative subpopulation.

### 3.3 Thresholds for high risk factors (Rounds 2–3)

In HR-positive patients, consensus was reached on thresholds independently indicative of high risk of recurrence for the following criteria: age, tumour grade, tumour size and number of positive nodes. In addition, thresholds indicative of high risk when present alongside other high-risk factors were agreed for tumour grade and number of positive nodes (Table 2; Table S3). No consensus was reached on appropriate thresholds for age or tumour size, when considered in combination with other factors.

**Table 2:** Results summary of outcomes and thresholds for HR-positive/HER2-negative and TN population (Rounds 2–3).

	HR-positive/HER2-negative^a^		TN^a^	
	Independently	In combination	Independently	In combination
**Age**	<35 years (71%)	<35 years (68%)	<35 years (62%)	<45 years (60%)
**Tumour size**	>5 cm (100%)	>5 cm (67%)	>2 cm (86%)	>2 cm (74%)
**Tumour grade**	Grade ≥3 (95%)	Grade ≥3 (84%)	Grade ≥3 (89%)	Grade ≥3 (79%)
**Number of positive lymph nodes**	≥4 (91%)	≥1 (73%)	≥1 (91%)	≥1 (95%)
**Tumour profiling tests**	Oncotype DX (89%)		N/A^b^	
**Risk prediction tools**	NHS Predict (95%)		RCB Index: RCB II (72%; 90%)	

**Footnotes:**^a^ Percentage agreement was calculated as a proportion of the number of respondents to a given question, i.e. excluding instances of ‘No response’. ^b^ Based on the responses to Round 1 (Fig. 3), the use of specific tumour profiling tests was only queried in HR-positive patients. **Abbreviations:** HER2: human epidermal growth factor receptor 2; HR: hormone receptor; RCB: Residual Cancer Burden; TN: triple-negative.

For patients with TN tumours, thresholds reached consensus, whether considered independently or in combination with other factors, for the following criteria: tumour size, tumour grade, number of positive nodes (Table 2; Table S4).There was no consensus reached on the appropriate threshold for age, either independently or in combination with other factors.

### 3.4 Tumour profiling tests and risk prediction tools (Rounds 1–3)

Only one tumour profiling test reached consensus for use in assessing risk of recurrence in HR-positive patients (Oncotype DX, 89%, 16/18) (Table 2; Table S5). Among the risk prediction tools, there was consensus that NHS Predict should be used to define high risk of recurrence in HR-positive/HER2-negative patients (95%, 18/19), and that the Residual Cancer Burden (RCB) Index should be used to define high risk of recurrence in TN patients (72%, 13/18). There was consensus that no additional tumour profiling tests or risk prediction tools should be used besides those already queried. As described above, consensus was not measured for statements concerning specific numerical thresholds for tumour profiling tests or risk prediction tools, which are presented in Table S5. Nevertheless, it is worth noting that none of the modal responses to these statements were chosen by 70% of panellists. RCB Index is measured in discrete scores, and there was consensus (90%, 18/20) that a threshold score of RCB II is indicative of high risk of recurrence.

## 4 Discussion

The results of this modified Delphi panel outline the key criteria typically used in current clinical practice to identify patients considered to be at high risk of recurrence of HER2-negative eBC after surgery. Additionally, thresholds suggested to be indicative of high recurrence risk, both independently and in combination with other high-risk factors, were elicited.

There was reasonable agreement among surveyed UK clinicians in the broad approach to identifying patients at high risk of disease recurrence, with many statements reaching high levels of consensus. When querying risk thresholds, tumour grade was the only factor for which agreed thresholds concurred across the HR-positive and TN populations (grade 3). For other factors, such as tumour size and number of positive nodes, participants generally indicated more stringent thresholds for defining high risk of recurrence in patients with HR-positive disease.

The results of the Delphi panel nevertheless highlighted some important areas of heterogeneity between respondents, including indicative risk thresholds for age and tumour size, as well as appropriate use of tumour profiling tests and risk prediction tools. Free-text responses suggested that the lack of consensus may have related to limited experience utilising these instruments, stemming from a lack of UK-wide availability, coupled with concerns around analytic validity and reliability in the case of Ki-67 status. Another key difficulty in obtaining consensus on these factors and thresholds is that different clinicians have fundamentally different conceptions of ‘high risk’, as evidenced in this study by the considerable heterogeneity of responses concerning appropriate timeframes and likelihoods of recurrence.

Given the multifactorial nature of recurrence risk assessment in eBC – including pathological, clinical and demographic factors – further complexity is introduced by the need to consider each factor within the context of the individual patient. Such context includes competing risks, preferences and attitudes, and available treatment options. This stands in contrast to the current UK Cancer Drugs Fund approvals system, which uses binary answers to questions about isolated risk factors to reach a decision, with no potential to use a combination of factors, account for the continuous scale of many risk factors, or gain special consideration if some factors are not assessable [25]. As such, it is important to continue developing available decision aid tools. Further research to understand the impact of factors such as ethnicity and body mass index may support the development of more individualised risk prediction models.

While the Delphi panel identified areas of consensus in the use of particular thresholds indicating a high risk of disease recurrence, it was beyond the scope of the exercise to assess specific combinations of factors and their interactions. Other potential limitations of this study include the relatively small sample size of clinicians and attrition between rounds. While beyond the scope of this study, the patient perspective can provide valuable insight when considering risk, which represents an important avenue for future research.

## 5 Conclusions

The expert consensus reached in this modified Delphi panel highlights that an integrated model is important in assessing recurrence risk in eBC and that definitions of high risk differ according to biological subtype. Although a comprehensive, standardised definition of high risk may be difficult to achieve in the context of relative risk/benefits for individual patients, the need for an objective definition based on absolute risk factors as described in this Delphi panel is critical given the increasing availability of targeted therapies for high-risk HER2-negative eBC. It is important that all patients with the potential to benefit from treatment are able to access these therapies easily and equitably, based on reliable and consistent risk assessments. Clinical consensus regarding high-risk eBC could inform clinical trial recruitment and stratification. It could also provide bodies such as NICE and NHS England with a valuable baseline evaluation and understanding of ‘high risk’ patients from the clinical community, leading to a positive impact on health economic evaluations.

## Author contributions

Substantial contributions to study conception and design: ERC; substantial contributions to analysis and interpretation of the data: ERC, JEA, JPB, DC, SAMcI, COM, AFCO, CP, FR, RR and SS; drafting the article or revising it critically for important intellectual content: ERC, JEA, JPB, DC, SAMcI, COM, AFCO, CP, FR, RR and SS; final approval of the version of the article to be published: ERC, JEA, JPB, DC, SAMcI, COM, AFCO, CP, FR, RR and SS.

## Delphi panellists

The following people completed all three rounds of the Delphi panel, contributing to the final results presented here: Jean Abraham, Annabel Borley, Jeremy Braybrooke, David Cameron, Mark Davies, Mike Dixon, Debashis Ghosh, Sarah Khan, Andreas Makris, Stuart McIntosh, Caroline Michie, Charlotte Moss, Mukesh Mukesh, Alicia Okines, Carlo Palmieri, Fharat Raja, Marcus Remer, Rebecca Roylance, Feng Yi Soh, Saiqa Spensley, Mark Tuthill and Lynda Wyld.

## Declaration of competing interest

The authors declare the following financial interests/personal relationships which may be considered as potential competing interests:

**ERC:** Consultant: 10.13039/100004325AstraZeneca, Lilly, 10.13039/100004336Novartis, 10.13039/100004319Pfizer, 10.13039/100004337Roche, Sanofi; Speaker fees: Novartis; Research grant: AstraZeneca; Educational grant: Daiichi Sankyo; Provision of research equipment: seca; Congress support: 10.13039/100004336Novartis, 10.13039/100004337Roche.

**JEA:** Research grant: AstraZeneca; Speaker fees: 10.13039/501100003769Eisai, 10.13039/100004319Pfizer.

**JPB:** Nothing to disclose.

**DC:** Consultant: 10.13039/100004325AstraZeneca, Exact Sciences, Lilly, 10.13039/100004336Novartis, Roche; Research funding: Exact Sciences, 10.13039/100004336Novartis, 10.13039/100004337Roche.

**SAMcI:** Consultant: Lilly, 10.13039/100009947MSD, Roche; Fees for non-CME services: BD; Congress support: Lilly, Roche; Research funding: 10.13039/100004336Novartis.

**COM:** Nothing to disclose.

**AFCO:** Research funding: 10.13039/100004319Pfizer, Roche; Consultant: 10.13039/100004325AstraZeneca, 10.13039/100004319Pfizer, 10.13039/100004337Roche, Seagen; Speaker fees: 10.13039/100004325AstraZeneca, 10.13039/100005564Gilead, Lilly, 10.13039/100004319Pfizer, Seagen; Congress support: AstraZeneca; Lilly.

**CP:** Consultant: 10.13039/100004325AstraZeneca, Daiichi Sankyo, Ellipses Pharma, Exact Sciences, 10.13039/100005564Gilead, Lilly, 10.13039/100004336Novartis, 10.13039/100004319Pfizer, Seagen; Research funding: Daiichi Sankyo, Exact Sciences, 10.13039/100004319Pfizer, Seagen; Congress support: 10.13039/100005564Gilead, 10.13039/100004337Roche.

**FR:** Consultant: 10.13039/100004325AstraZeneca, Daiichi Sankyo, 10.13039/100005564Gilead, Lilly, 10.13039/100009947MSD, 10.13039/100004336Novartis, 10.13039/100004319Pfizer, Roche; Congress support: 10.13039/100004325AstraZeneca, 10.13039/100009947MSD, 10.13039/100004336Novartis, 10.13039/100004337Roche.

**RR:** Consultant: 10.13039/100004325AstraZeneca, Daiichi Sankyo, G1 Therapeutics, IQVIA, Lilly, Pfizer; Congress support: BMS, Daiichi Sankyo, Roche; Grants to institution: 10.13039/501100000272NIHR.

**SS:** Consultant: Lilly, Pfizer.

## Footnotes

a The name erbB-2 receptor tyrosine kinase 2 (ERBB2) is increasingly preferred, though HER2 is still commonly used in clinical practice.
