# Supplementary material for: Expert UK consensus on the definition of high risk of recurrence in HER2-negative early breast cancer: A modified Delphi panel
Source: Breast. 2023 Sep 17;72:103582. doi: 10.1016/j.breast.2023.103582 (PMC10539921; doi:10.1016/j.breast.2023.103582)
Supplement: Multimedia component 1 [file mmc1.pdf]

Expert UK consensus on the definition of high risk of recurrence in HER2-negative early breast cancer: a modified Delphi panel

E. R. Copson,[a] J. E. Abraham,[b,c] J. P. Braybrooke,[d] D. Cameron,[e] S. A. McIntosh,[f] C. O. Michie,[e] A. F. C. Okines,[g] C. Palmieri,[h,i] F. Raja,[j,k] R. Roylance,[j,l] S. Spensley,[m] on behalf of the Delphi panellists

[a]Cancer Sciences Academic Unit, University of Southampton, Southampton, UK; [b]Precision Breast Cancer Institute, Department of Oncology, University of Cambridge, Cambridge, UK; [c]Cambridge University Hospitals NHS Foundation Trust, Cambridge, UK; [d]University Hospitals Bristol and Weston NHS Foundation Trust, Bristol, UK; [e]Edinburgh Cancer Centre, Western General Hospital, Edinburgh, UK; [f]Patrick G Johnston Centre for Cancer Research, Queen's University Belfast, Belfast, UK; [g]The Royal Marsden NHS Foundation Trust, London, UK; [h]The Clatterbridge Cancer Centre NHS Foundation Trust, Liverpool, UK; [i]Department of Molecular and Clinical Cancer Medicine, University of Liverpool, Liverpool, UK; [j]University College London Hospitals NHS Foundation Trust, London, UK; [k]North Middlesex University Hospital, North Middlesex University Hospital NHS Trust, London, UK; [l]NIHR University College London Hospitals Biomedical Research Centre, London, UK; [m]Musgrove Park Hospital, Somerset NHS Foundation Trust, Taunton, UK

SUPPLEMENTARY MATERIALS

Targeted Literature Review Methodology

The targeted literature review was conducted in February 2022 to collate current definitions for patients at high risk of recurrence in the eBC population in the peer-reviewed and grey (non-peer reviewed) literature sources, including websites of key professional bodies, clinical trial registries and congress proceedings. Due to the targeted nature of the literature review, results were not expected to comprehensively capture all relevant literature, but instead aimed to identify trends in the definition of patients at high risk of recurrence in the HER2-negative eBC population in clinical guidelines, clinical trials, primary research articles of recurrence risk and non-primary research publications including consensus statements, literature reviews, commentaries or opinion pieces.

Literature searches were carried out in MEDLINE and Embase (simultaneously via Ovid SP), using key terms for high risk of recurrence in the eBC population. Searches were date-limited from 1st January 2012 to 7th February 2022. Clinical trial registry searches using the ClinicalTrials.gov platform (https://clinicaltrials.gov/ct2/home) were carried out to identify eligibility criteria used in recruitment for clinical trials conducted in the high-risk eBC indication. Websites of the following key professional bodies were also searched to identify any relevant treatment guidelines or consensus statements relevant to the definition of high risk in eBC:

- American Society of Clinical Oncology (ASCO) (https://www.asco.org/)
- European Organisation for Research and Treatment of Cancer (EORTC) (https://www.eortc.org/)
- European Society for Medical Oncology (ESMO) (https://www.esmo.org/)
- National Cancer Research Institute (NCRI) (https://www.ncri.org.uk/)
- The National Institute for Health and Care Excellence (NICE) (https://www.nice.org.uk/)
- The Royal College of Radiologists (RCR) (https://www.rcr.ac.uk/)
- UK Breast Cancer Group (UKBCG) (https://ukbcg.org/)

Table S1. Responses to criterion-based statements: risk factors (Rounds 1–2)

Statement	Delphi round	Outcome	Response breakdown (%, n/N)[a]
In the HER2-negative eBC population, the following criterion should be used in clinical practice to define high risk of recurrence (in all patients or at least one subgroup):			
Age	Round 1	**Agreement**	**Yes (82%, 23/28)**<br>No (18%, 5/28)<br>*No response: 1*
Menopausal status	Round 1	No consensus reached	Yes (44%, 12/27)<br>No (56%, 15/27)<br>*No response: 2*
Tumour size	Round 1	**Agreement**	**Yes (93%, 26/28)**<br>No (7%, 2/28)<br>*No response: 1*
Tumour grade	Round 1	**Agreement**	**Yes (100%, 28/28)**<br>No (0)<br>*No response: 1*
Number of positive lymph nodes	Round 1	**Agreement**	**Yes (96%, 27/28)**<br>No (4%, 1/28)<br>*No response: 1*
pCR/residual disease	Round 1	**Agreement**	**Yes (100%, 28/28)**<br>No (0)<br>*No response: 1*
Biomarker(s) (e.g. Ki-67)	Round 1	**Agreement**	**Yes (71%, 20/28)**<br>No (29%, 8/28)<br>*No response: 1*
	Round 2	**Agreement**	**Yes (88%, 21/24)**<br>No (12%, 3/24)<br>*No response: 0*
Germline *BRCA1/2* mutation status[b]	Round 1	No consensus reached	Yes (59%, 16/27)<br>No (41%, 11/27)<br>*No response: 2*
	Round 2	**Agreement**	Yes (26%, 5/19)<br>**No (74%, 14/19)**<br>*No response: 5*
Tumour profiling test(s)	Round 1	**Agreement**	**Yes (100%, 27/27)**<br>No (0)<br>*No response: 2*
Risk prediction tool(s)	Round 1	**Agreement**	**Yes (100%, 27/27)**<br>No (0)<br>*No response: 2*
Inflammatory breast cancer	Round 1	**Agreement**	**Yes (100%, 27/27)**<br>No (0)<br>*No response: 2*
Any other criterion not listed previously	Round 1	No consensus reached	Yes (33%, 9/27)<br>No (67%, 18/27)<br>*No response: 2*

Footnotes: [a] Percentage agreement was calculated as a proportion of the number of respondents to a given question (N), i.e. excluding instances of 'No response'. [b] This criterion was referred to as 'Breast cancer gene (BRCA) status' in Round 1. **Abbreviations:** *BRCA1/2*: breast cancer susceptibility gene 1/2; eBC: early breast cancer; HER2: human epidermal growth factor receptor 2; pCR: pathologic complete response; TN: triple-negative.

Table S2. Responses to criterion-based statements: patient populations (Round 1)

**Statement**	**Outcome**	**Response breakdown (%, n/N)[a]**
In which patients should the following criterion be used in clinical practice to define high risk of recurrence?[b]		
Age	**Agreement**	**All HER2-negative (83%, 19/23)**<br>HR-positive only (9%, 2/23)<br>TN only (9%, 2/23)<br>*No response: 5*
Tumour size	**Agreement**	**All HER2-negative (100%, 26/26)**<br>HR-positive only (0)<br>TN only (0)<br>*No response: 2*
Tumour grade	**Agreement**	**All HER2-negative (93%, 26/28)**<br>HR-positive only (7%, 2/28)<br>TN only (0)<br>*No response: 0*
Number of positive lymph nodes	**Agreement**	**All HER2-negative (96%, 26/27)**<br>HR-positive only (0)<br>TN only (4%, 1/27)<br>*No response: 1*
pCR/residual disease	No consensus reached	All HER2-negative (46%, 13/28)<br>HR-positive only (0)<br>TN only (54%, 15/28)<br>*No response: 0*
Biomarker(s) (e.g. Ki-67)	No consensus reached	All HER2-negative (55%, 11/20)<br>HR-positive only (45%, 9/20)<br>TN only (0)<br>*No response: 8*
Tumour profiling test(s)	**Agreement**	All HER2-negative (11%, 3/27)<br>**HR-positive only (89%, 24/27)**<br>TN only (0)<br>*No response: 0*
Risk prediction tool(s)	**Agreement**	**All HER2-negative (78%, 21/27)**<br>HR-positive only (22%, 6/27)<br>TN only (0)<br>*No response: 0*
Inflammatory breast cancer	**Agreement**	**All HER2-negative (93%, 25/27)**<br>HR-positive only (0)<br>TN only (7%, 2/27)<br>*No response: 0*

Footnotes: [a] Percentage agreement was calculated as a proportion of the number of respondents to a given question (N), i.e. excluding instances of 'No response'. [b] Only panellists who selected 'Yes' to the corresponding statements querying the use of specific criteria were invited to respond; this is reflected in the varying N + 'No response' sums for different criteria. Results for criteria having failed to reach consensus (see **Table A1**) are not displayed. **Abbreviations:** HER2: human epidermal growth factor receptor 2; HR: hormone receptor; pCR: pathologic complete response; TN: triple-negative.

Table S3. Responses to threshold-based statements concerning HR-positive/HER2-negative population (Rounds 2–3)

Statement	Delphi round	Outcome	Response breakdown (%, n/N)[a]
Please indicate which threshold you would consider to be indicative of high risk of recurrence in the HR-positive/HER2-negative population for the following criterion:			
Age (when considered independently)	Round 2	No consensus reached	<35 years (55%, 11/20)<br><40 years (25%, 5/20)<br><45 years (5%, 1/20)<br>Other (0)<br>None – requires combination of factors (15%, 3/20)<br>*No response: 4*
	Round 3	**Agreement**	**<35 years (71%, 15/21)**<br><40 years (29%, 6/21)<br>*No response: 1*
Age (when considered in combination with other high-risk factors)	Round 2	No consensus reached	<35 years (47%, 9/19)<br><40 years (26%, 5/19)<br><45 years (11%, 2/19)<br>Other (0)<br>None – always independently sufficient (16%, 3/19)<br>*No response: 5*
	Round 3	No consensus reached	<35 years (68%, 15/22)<br><40 years (32%, 7/22)<br>*No response: 0*
Tumour size (independent)	Round 2	No consensus reached	>2 cm (21%, 4/19)<br>>5 cm (53%, 10/19)<br>Other (5%, 1/19)<br>None – requires combination of factors (21%, 4/19)<br>*No response: 5*
	Round 3	**Agreement**	>2 cm (0)<br>>5 cm (100%, 20/20)<br>*No response: 2*
Tumour size (combined)	Round 2	No consensus reached	>2 cm (37%, 7/19)<br>>5 cm (42%, 8/19)<br>Other (5%, 1/19)<br>None – always independently sufficient (16%, 3/19)<br>*No response: 5*
	Round 3	No consensus reached	>2 cm (33%, 7/21)<br>>5 cm (67%, 14/21)<br>*No response: 1*
Tumour grade (independent)	Round 2	**Agreement**	Grade ≥1 (0)<br>Grade ≥2 (0)<br>**Grade ≥3 (95%, 18/19)**<br>Other (0)<br>None – requires combination of factors (5%, 1/19)<br>*No response: 5*
Tumour grade (combined)	Round 2	**Agreement**	Grade ≥1 (0)<br>Grade ≥2 (16%, 3/19)<br>**Grade ≥3 (84%, 16/19)**<br>Other (0)

			None – always independently sufficient (0)<br>*No response: 5*
Number of positive lymph nodes (independent)	Round 2	No consensus reached	≥1 (21%, 4/19)<br>≥2 (0)<br>≥3 (11%, 2/19)<br>≥4 (53%, 10/19)<br>Other (11%, 2/19)<br>None – requires combination of factors (5%, 1/19)<br>*No response: 5*
	Round 3	**Agreement**	≥1 (9%, 2/22)<br>**≥4 (91%, 20/22)**<br>*No response: 0*
Number of positive lymph nodes (combined)	Round 2	No consensus reached	≥1 (47%, 9/19)<br>≥2 (0)<br>≥3 (16%, 3/19)<br>≥4 (11%, 2/19)<br>Other (5%, 1/19)<br>None – always independently sufficient (21%, 4/19)<br>*No response: 5*
	Round 3	**Agreement**	**≥1 (73%, 16/22)**<br>≥3 (27%, 6/22)<br>*No response: 0*

Footnote: [a] Percentage agreement was calculated as a proportion of the number of respondents to a given question (N), i.e. excluding instances of 'No response'. **Abbreviations:** HER2: human epidermal growth factor receptor 2; HR: hormone receptor.

Table S4. Responses to threshold-based statements concerning TN population (Rounds 2–3)

Statement	Delphi round	Outcome	Response breakdown (%, n/N)[a]
Please indicate which threshold you would consider to be indicative of high risk of recurrence in the TN population for the following criterion:			
Age (when considered independently)	Round 2	No consensus reached	<35 years (26%, 5/19)<br><40 years (16%, 3/19)<br><45 years (32%, 6/19)<br>Other (5%, 1/19)<br>None – requires combination of factors (21%, 4/19)<br>*No response: 5*
	Round 3	No consensus reached	<35 years (62%, 13/21)<br><40 years (24%, 5/21)<br><45 years (14%, 3/21)<br>*No response: 1*
Age (when considered in combination with other high-risk factors)	Round 2	No consensus reached	<35 years (22%, 4/18)<br><40 years (22%, 4/18)<br><45 years (44%, 8/18)<br>Other (6%, 1/18)<br>None – always independently sufficient (6%, 1/18)<br>*No response: 6*
	Round 3	No consensus reached	<35 years (35%, 7/20)<br><40 years (5%, 1/20)<br><45 years (60%, 12/20)<br>*No response: 2*
Tumour size (independent)	Round 2	No consensus reached	>2 cm (68%, 13/19)<br>>5 cm (11%, 2/19)<br>Other (5%, 1/19)<br>None – requires combination of factors (16%, 3/19)<br>*No response: 5*
	Round 3	**Agreement**	**>2 cm (86%, 18/21)**<br>>5 cm (14%, 3/21)<br>*No response: 1*
Tumour size (combined)	Round 2	**Agreement**	**>2 cm (74%, 14/19)**<br>>5 cm (0)<br>Other (16%, 3/19)<br>None – always independently sufficient (11%, 2/19)<br>*No response: 5*
Tumour grade (independent)	Round 2	**Agreement**	Grade ≥1 (0)<br>Grade ≥2 (0)<br>**Grade ≥3 (89%, 17/19)**<br>Other (0)<br>None – requires combination of factors (11%, 2/19)<br>*No response: 5*
Tumour grade (combined)	Round 2	**Agreement**	Grade ≥1 (5%, 1/19)<br>Grade ≥2 (11%, 2/19)<br>**Grade ≥3 (79%, 15/19)**<br>Other (0)<br>None – always independently sufficient (5%, 1/19)<br>*No response: 5*

Number of positive lymph nodes (independent)	Round 2	No consensus reached	≥1 (53%, 10/19)<br>≥2 (11%, 2/19)<br>≥3 (5%, 1/19)<br>≥4 (21%, 4/19)<br>Other (0)<br>None – requires combination of factors (11%, 2/19)<br>*No response: 5*
	Round 3	**Agreement**	**≥1 (91%, 20/22)**<br>≥4 (9%, 2/22)<br>*No response: 0*
Number of positive lymph nodes (combined)	Round 2	No consensus reached	≥1 (68%, 13/19)<br>≥2 (5%, 1/19)<br>≥3 (5%, 1/19)<br>≥4 (11%, 2/19)<br>Other (5%, 1/19)<br>None – always independently sufficient (11%, 2/19)<br>*No response: 5*
	Round 3	**Agreement**	**≥1 (95%, 21/22)**<br>≥4 (5%, 1/22)<br>*No response: 0*

Footnote: [a] Percentage agreement was calculated as a proportion of the number of respondents to a given question (N), i.e. excluding instances of 'No response'. **Abbreviations:** TN: triple-negative.

Table S5. Responses to questions concerning the use of specific tumour profiling tests and risk prediction tools (Rounds 2–3)

Statement	Delphi round	Outcome	Mode (%, n/N)[a]
The following assay should be used in clinical practice to define high risk of recurrence in the HR-positive/HER2-negative population:			
EndoPredict	Round 2	No consensus reached	Yes (53%, 10/19)
Oncotype DX	Round 2	**Agreement**	**Yes (89%, 16/18)**
Prosigna	Round 2	No consensus reached	Yes (63%, 12/19)
Any other?	Round 2	**Agreement**	**No (83%, 15/18)**
For the following assay, please indicate the threshold score (≥) which you would consider indicative of high risk of recurrence at 10 years in the HR-positive/HER2-negative population:			
EndoPredict	Round 2	*Consensus not measured*	3.3 (50%, 4/8)
Oncotype DX	Round 2	*Consensus not measured*	26 (50%, 7/14)
Prosigna	Round 2	*Consensus not measured*	61 (50%, 4/8)
The following tool should be used in clinical practice to define high risk of recurrence in the specified population:			
NHS Predict (HR-positive/HER2-negative)	Round 2	**Agreement**	**Yes (95%, 18/19)**
NHS Predict (TN)	Round 2	No consensus reached	Yes (61%, 11/18)
RCB Index (HR-positive/HER2-negative)	Round 2	No consensus reached	Yes (50%, 9/18)<br>No (50%, 9/18)
RCB Index (TN)	Round 2	**Agreement**	**Yes (72%, 13/18)**
Any other? (HR-positive/HER2-negative and/or TN)	Round 2	**Agreement**	**No (94%, 17/18)**
For the following tool, please indicate the difference in estimated 10-year overall survival rate with breast cancer (compared with anticipated 10-year overall survival if deaths with breast cancer were excluded) which would constitute a high risk of disease recurrence in the specified population:			
NHS Predict (HR-positive/HER2-negative)	Round 2	*Consensus not measured*	5%-point reduction in survival (47%, 8/17)
NHS Predict (TN)	Round 2	*Consensus not measured*	5%-point reduction in survival (45%, 5/11)
For the following assay, please indicate the threshold score (≥) which you would consider indicative of high risk of recurrence at 10 years in the specified population:			
RCB Index (HR-positive/HER2-negative)	Round 2	**Agreement**	**RCB II (75%, 6/8)**
RCB Index (TN)	Round 2	No consensus reached	RCB II (67%, 8/12)
	Round 3	**Agreement**	**RCB II (90%, 18/20)**

Footnote: [a] 'No response' rates not shown; Rounds 2 and 3 had 24 and 22 overall respondents, respectively. Percentage agreement was calculated as a proportion of the number of respondents to a given question (N), i.e. excluding instances of 'No response'. **Abbreviations:** HER2: human epidermal growth factor receptor 2; HR: hormone receptor; RCB: Residual Cancer Burden; TN: triple-negative.
